# Influence of HLA-B27 on the Ankylosing Spondylitis phenotype: results from the REGISPONSER database

**DOI:** 10.1186/s13075-018-1724-7

**Published:** 2018-10-03

**Authors:** Marta Arévalo, Jordi Gratacós Masmitjà, Mireia Moreno, Joan Calvet, Cristobal Orellana, Desirée Ruiz, Carmen Castro, Pilar Carreto, Marta Larrosa, Eduardo Collantes, Pilar Font, REGISPONSER group

**Affiliations:** 1 Rheumatology Department, Consorci Corporació Sanitària Parc Taulí, Institut d’Investigació i Innovació Parc Taulí I3PT, Universitat Autònoma de Barcelona, Parc Taulí s/n, 08208 Sabadell, Barcelona Spain; 2 Rheumatology Department, Hospital General Universitario Reina Sofía/IMIBIC/Universidad de Córdoba, Córdoba, Spain

**Keywords:** Ankylosing Spondylitis, HLA-B27, Structural damage, Phenotype, Uveitis

## Abstract

**Objective:**

To assess HLA-B27 influence on the clinical phenotype of Ankylosing Spondylitis (AS) patients.

**Method:**

An observational, cross-sectional and descriptive study of AS patients from the Spanish REGISPONSER database was performed. Demographic, clinical, disease activity (Bath Ankylosing Spondylitis Disease Activity Index (BASDAI), Bath Ankylosing Spondylitis Functional Index (BASFI), erythrocyte sedimentation rate (ESR), and C-reactive protein (CRP)), and radiographic data (Bath Ankylosing Spondylitis Radiology Index (BASRI) score) were compared regarding HLA-B27 status. A univariate and multivariate analysis was performed to identify variables independently related to the presence of HLA-B27.

**Results:**

Data from 1235 patients (74.8% male) were analyzed; 1029 were HLA-B27 positive (83%). HLA-B27-positive patients showed higher family aggregation and an earlier onset of disease compared with those who were HLA-B27 negative. HLA-B27-negative patients presented statistically higher BASDAI and BASFI scores and higher prevalence of arthritis, dactylitis, and extra-articular manifestations (psoriasis and inflammatory bowel disease (IBD)) but not anytime uveitis compared with those who were HLA-B27 positive.

In the multivariate analysis, family history (odds ratio (OR) 2.10, 95% confidence interval (CI) 1.27–3.49), younger age at diagnosis (OR 0.97, 95% CI 0.96–0.98), presence of peripheral arthritis (OR 0.53, 95% CI 0.32–0.89), dactylitis (OR 0.16, 95% CI 0.05–0.56), psoriasis (OR 0.45, 95% CI 0.26–0.78), and IBD (OR 0.22, 95% CI 0.12–0.40) were the main variables independently related to the presence or not of HLA-B27.

**Conclusion:**

In Caucasian AS patients, the presence of HLA-B27 is related to an earlier disease onset and higher family aggregation. Absence of HLA-B27 is related to a higher frequency of peripheral arthritis, dactylitis, and extra-articular manifestations. Being HLAB27 positive is not related to a higher burden of disease or anytime uveitis.

## Background

Axial Spondyloarthritis (AxSpA) includes an heterogeneous group of rheumatisms characterized by their strong association with HLA-B27 and axial skeleton involvement. Ankylosing Spondylitis (AS) is the main disease of this group and is clinically defined by inflammatory back pain, but it can also involve other sites such as peripheral arthritis, enthesitis, dactylitis, and extra-articular manifestations as uveitis, psoriasis, and inflammatory bowel disease (IBD). Frequently, disease onset occurs in patients 20–30 years of age and, if no effective treatment is given, it can lead to severe disability in nearly a third of individuals [1]. Its prevalence is around 0.2–0.3% depending on the geographical distribution of HLA-B27 [2, 3]. HLA-B27, since its discovery in 1973 [4], constitutes the main genetic factor related to disease etiopathogenesis. However, nearly 10–20% of patients with defined AS do not carry HLA-B27, which increases to 40% when analyzing nonradiographic axial spondyloarthritis (nrAxSpA) [5]. Previous studies suggest a relationship between HLA-B27 and axial manifestations including structural progression in early AxSpA [5]. Moreover, a younger age of onset, more family history [5, 6], and less prevalence of psoriasis and IBD [5] have also been reported. Nevertheless, there are few studies evaluating the role of HLA-B27 in defined AS patients [7–9], with some of them focused on radiographic progression [10–12] and reporting controversial results [13]. The main objective of the present study was to evaluate the HLA-B27 influence on the clinical expression of defined AS patients. For this purpose, we reviewed data from the REGISPONSER database [14] which includes more than 1000 AS patients, of whom about 20% are HLA-B27 negative.

## Material and methods

**Fig. 1:**
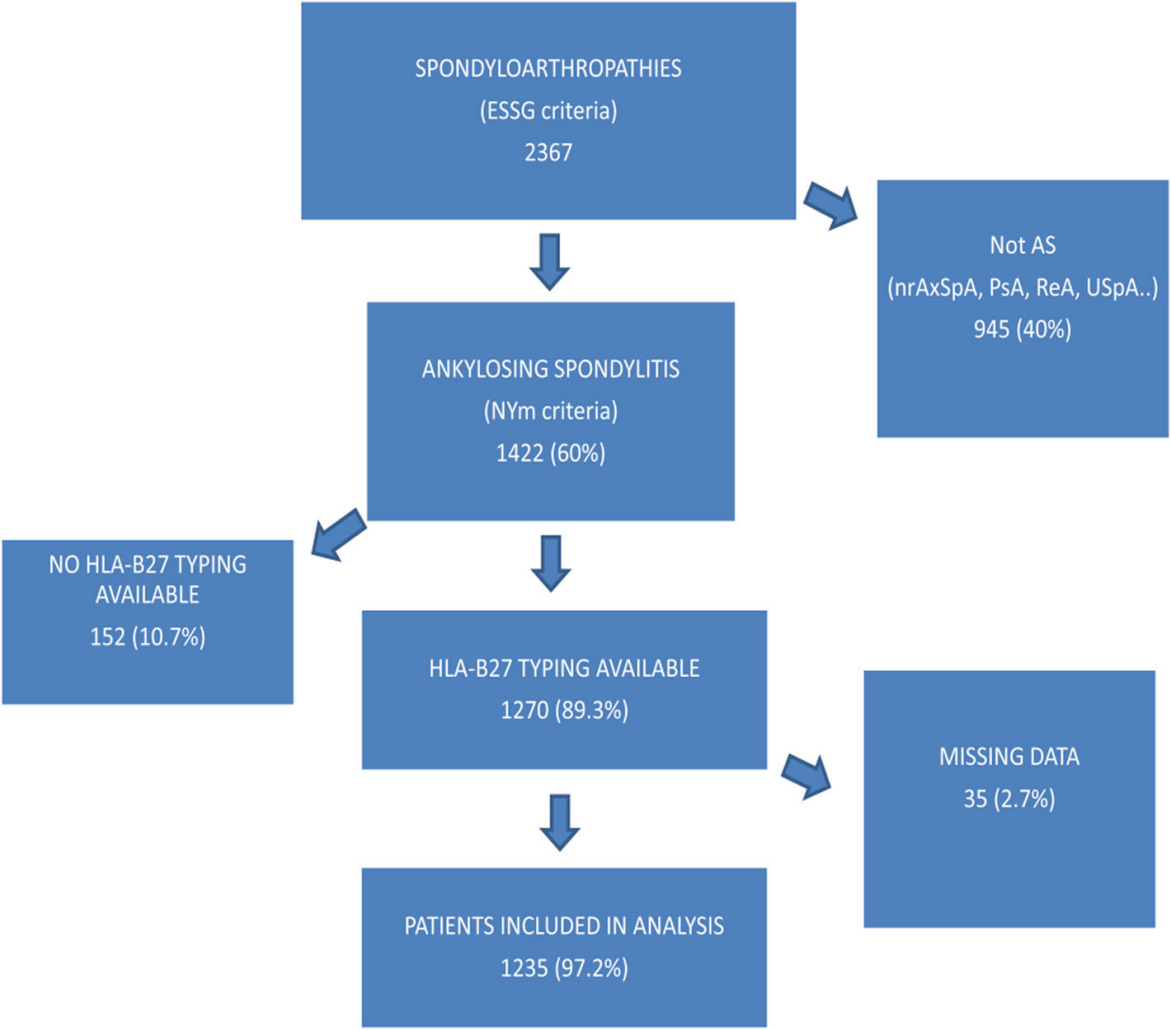
Flow chart of patients included in the study from the REGISPONSER database. AS Ankylosing Spondylitis, ESSG European Spondyloarthropathy Study Group, nrAxSpA nonradiographic axial spondyloarthritis, NYm New York modified, PsA psoriatic arthritis, ReA reactive arthritis, USpA undifferentiated spondyloarthritis

This is a comparative, cross-sectional study including all patients fulfilling AS New York modified criteria from the REGISPONSER. REGISPONSER is a Spanish registry that includes 2367 patients who fulfilled European Spondyloarthropathy Study Group (ESSG) criteria for spondyloarthritis. Of these, 1422 had radiographic sacroiliitis as per New York modified criteria, with 1270 of these having HLA-B27 typing available, and finally 1235 of these having no data of interest missing (Fig. 1). Recruitment of patients started in March 2004 and finished in March 2007. All patients included signed an informed consent form, and the project was approved by the ethical committee of all participant hospitals. More information about the methodology and data inclusion are detailed in a previous publication by Collantes et al. [14].

The study included the following clinical and biological variables: age (in years), gender, the presence of HLA-B27, age at disease onset (in years), disease duration (in years), diagnosis delay (in years), axial symptoms, peripheral involvement (peripheral arthritis, coxitis, enthesitis, and dactylitis), extra-articular manifestations confirmed by a specialist (uveitis, IBD, psoriasis, palmoplantar pustulosis, balanitis, prostatitis), clinical disease activity (Bath Ankylosing Spondylitis Disease Activity Index (BASDAI), 0–10 cm), clinical disability (Bath Ankylosing Spondylitis Functional Index (BASFI), 0–10 cm), biological disease activity (erythrocyte sedimentation rate (ESR) and C-reactive protein (CRP)), and structural damage (Bath Ankylosing Spondylitis Radiology Index (BASRI)) assessed by a local radiologist and confirmed by an experienced rheumatologist.

### Statistical analysis

A descriptive analysis of the variables was performed using absolute and relative frequencies for qualitative variables and mean with standard deviation for the quantitative variables. A 95% confidence interval (CI) was calculated. For bivariate analysis the Chi-squared test was used, and the Student *t* test was used for independent data. Finally, a multiple logistic regression model was performed including the following variables: gender, age, age at onset, disease duration, signs and symptoms at onset and during follow-up, peripheral and extra-articular manifestations, BASDAI, BASFI, BASRI, ESR, and CRP. The degree of association was expressed as the odds ratio (OR) and Cornfield confidence interval was established at 95% using Wald statistics. Variables with *p* ≥ 0.15 were suppressed from the model one by one (backward stepwise procedure). Comparison between the reduced model and the one including suppressed variables was made using the likelihood ratio test (G statistic). A scale of continuous variables was evaluated with the Bow Tidwell test. Possible interactions between variables were studied as possible confounding factors, considering them if the percentage change of coefficients was higher than 20%. The Hosmer Lemeshow statistic was performed to assess the goodness of fit. All contrasts were bilateral and considered significant when *p* < 0.05. Data were collected, processed, and analyzed using SPSS v.17.

## Results

Data from 1235 patients were collected, with 1029 (83.3%) patients being HLA-B27 positive and 206 (17.7%) patients being HLA-B27 negative. Male gender was observed in 924 (74.8%) patients with no differences between both groups (74.7% of males in the HLA-B27-positive group vs. 75.2% in the HLA-B27-negative group; not significant). HLA-B27-positive patients showed a higher family aggregation (22.1% vs 12%; *p* = 0.002), younger age (47.9 vs 50.4 years; *p* = 0.012), and younger age at symptom onset (26.2 vs 30.6 years; *p* < 0.001) and at diagnosis (33.9 vs 39 years; *p* < 0.001) compared with HLA-B27-negative patients. However, the absence of HLA-B27 did not lead to a greater diagnosis delay (7.8 vs. 8.5 years; not significant). Analyzing disease activity, HLA-B27-negative patients expressed a higher disease activity measured by BASDAI (4.1 ± 2.3 vs 4.4 ± 2.4; *p* = 0.047) and higher clinical disability measured by BASFI (3.8 ± 2.7 vs 4.3 ± 2.9; *p* = 0.005) compared with HLA-B27-positive patients. However, no differences were observed between groups regarding the biological markers analyzed (ESR and CRP), nor in structural damage expressed by BASRI (7.2 vs 7.4; not significant). Moreover, HLA-B27-negative patients showed a higher prevalence of peripheral arthritis (15.4% vs 21.8%; *p* = 0.023), dactylitis (0.8% vs 3.9%; *p* = 0.001), and extra-articular manifestations, especially psoriasis (5.9% vs 14.6%; *p* < 0.001) and IBD (3.6% vs 11.7%; *p* < 0.001) compared with HLA-B27-positive patients. On the other hand, this study did not find any statistical differences regarding axial clinical disease expression (neck pain, low back pain, and sacroiliac joint symptoms), the presence of enthesitis (present in 6.1% of subjects) or uveitis (22.4% in HLA-B27-positive and 19.4% in HLA-B27-negative patients; not significant).

In the multivariate analysis, family history (OR 2.10, 95% CI 1.27–3.49; *p* = 0.004), younger age at diagnosis (OR 0.97, 95% CI 0.96–0.98; *p* < 0.001), and the presence of peripheral arthritis (OR 0.53, 95% CI 0.32–0.89; *p* = 0.016), dactylitis (OR 0.16, 95% CI 0.05–0.56; *p* = 0.004), psoriasis (OR 0.45, 95% CI 0.26–0.78; *p* = 0.005), and IBD (OR 0.22, 95% CI 0.12–0.40; *p* < 0.001) were the main variables independently related to the presence or not of HLA-B27. All results are shown in Table 1.

**Table 1:** Univariate and multivariate analysis between the HLA-B27-positive and HLA-B27-negative groups

	B27 negative		B27 postive		Univariate analysis	Multivariate analysis		
	*n* (= 206)	Mean ± SD or %^a^	*n* (= 1029)	Mean ± SD or %^a^	*p*	OR	95% CI	*p*
Men	155	75.2	769	74.7	NS			
Family history	22	12	216	22.1	0.002	2.1	1.27–3.49	0.004
Age (years), mean ± SD	206	50,4 ± 12,9	1025	47,9 ± 13	0,012			
Age at onset (years), mean ± SD	204	30.6 ± 12.3	1010	26.2 ± 9.9	< 0.001			
Age at diagnosis (years), mean ± SD	199	39 ± 12.3	1002	33.9 ± 11.5	< 0.001	0.97	0.96–0.98	< 0.001
Disease duration since first symptom (years), mean ± SD	204	19.8 ± 12.8	1013	21.9 ± 13.1	0.038			
Diagnosis delay (years), mean ± SD	198	8.5 ± 9.6	993	7.8 ± 9.2	NS			
Disease duration since diagnosis (years), mean ± SD	199	11.3 ± 9.3	1004	14 ± 10.6	0.001			
Axial manifestations								
Cervicalgia	28	13.6	103	10	NS			
Lumbalgia	148	71.8	741	72	NS			
Sacroiliac syndrome	85	41.3	435	42.3	NS			
Peripheral manifestations								
Peripheral arthritis	45	21.8	158	15.4	0.023	0.53	0.32–0.89	0.016
Coxitis	6	2.9	32	3.1	NS			
Enthesitis	10	4.9	65	6.3	NS			
Dactylitis	8	3.9	8	0.8	0.001	0.16	0.05–0.56	0.004
Extra-articular manifestations								
Psoriasis	30	14.6	61	5.9	< 0.001	0.45	0.26–0.78	0.005
Inflammatory bowel disease	24	11.7	37	3.6	< 0.001	0.22	0.12–0.4	< 0.001
Urethritis, cervicitis, diarrhea	4	1.9	7	0.7	NS			
Anterior uveitis	40	19.4	230	22.4	NS			
Palmoplantar pustulosis	6	2.9	4	0.4	0.002	0.12	0.02–0.64	0.013
Balanitis	4	1.9	4	0.4	0.022			
Prostatitis	1	0.5	7	0.7	NS			
BASRI score, mean ± SD	194	7.4 ± 4.1	974	7.2 ± 3.9	NS			
BASDAI score, mean ± SD	206	4.4 ± 2.4	1029	4.1 ± 2.3	0.047			
BASFI score, mean ± SD	206	4.3 ± 2.9	1028	3.8 ± 2.7	0.005	0.94	0.88–1	0.037
ESR (mm/1 h), mean ± SD	174	19 ± 15.98	977	18.3 ± 16.3	NS			
CRP (mg/L), mean ± SD	174	10.2 ± 17.7	975	9.2 ± 13.7	NS			

*BASDAI* Bath Ankylosing Spondylitis Disease Activity Index, *BASFI* Bath Ankylosing Spondylitis Functional Index, *BASRI* Bath Ankylosing Spondylitis Radiology Index, *CRP* C-reactive protein, *ESR* erythrocyte sedimentation rate, *NS* not significant

^a^ For qualitative variables as percentage (%) and for quantitative variables as mean ± standard deviation (SD)

## Discussion

This is the most extensive study performed comparing clinical characteristics of Caucasian AS patients regarding HLA-B27. The study confirms the association of HLA-B27 with earlier disease onset and family aggregation. Moreover, the study proves that the absence of HLA-B27 in AS patients is related to a higher frequency of peripheral arthritis, psoriasis, and IBD. Finally, our data do not support the relationship between HLA-B27 and the severity of axial structural damage in AS patients.

The prevalence of HLA-B27 in our study reaches 83%, similar to that previously reported in AS patients [2]. We observed a male:female ratio of 3:1, comparable with that shown in previous studies in AS patients regardless of the presence of HLA-B27 [15], but clearly different from the previously reported gender ratio in nrAxSpA patients where there is not a male predominance [5, 6]. Our results are also not in accordance with those previously published by Yang et al. who reported a clear male predominance in AS patients associated with the presence of HLA-B27 [9]. However, that study was performed in an Asian population, and race differences need to be considered.

Our study, using the BASRI score, did not show statistical differences regarding axial structural damage in HLA-B27-positive AS patients compared with HLA-B27-negative patients. In this sense, our data are in accordance with those reported in the GESPIC cohort [6]. Previous data regarding the influence of HLA-B27 on the progression and extent of radiographic axial damage in AS patients are controversial due to heterogeneity in published series and the methods used when measuring structural damage [9–13]. In the DESIR cohort, the authors observed higher radiographic damage in early AxSpA patients related to the presence of HLA-B27, but only in the sacroiliac joints [5]. No differences in radiographic spine damage measured by the modified Stoke Ankylosing Spondylitis Spine Score (mSASSS) were found. When looking at studies including definite AS patients, our results differ from those reported by Yang et al. [9], who found a higher radiographic extent of structural damage in Asian AS patients and also in the HLA-B27-positive compared to the HLA-B27-negative patients. We do not have any precise explanation for these observed differences, but we cannot exclude some unknown variables related to the genetic and/or environmental factors when comparing different genetic populations. Moreover, since male gender seems to be a strong predictive factor for radiographic damage [6, 16] and the male ratio in HLA-B27-positive AS patients was clearly higher in the study by Yang et al. compared with our study [9], we need to consider this variable as a potential explanation for the differences observed between both studies. Finally, it is also interesting to remark that disease duration was significantly higher in HLA-B27-positive AS patients than the other group. Given that disease duration is one of the main factors related to structural damage, the data reported here do not support a major role of HLA-B27 in the extension of the structural damage in defined AS patients.

Our data appear to confirm previous studies suggesting that HLA-B27 is associated with a younger disease onset and greater family aggregation in AS patients [9, 13]. Similar results were also described in early forms of HLA-B27-positive patients from the DESIR [5] and GESPIC [6] cohorts, in such a way that HLA-B27 seems to anticipate the clinical manifestations and disease onset. Although HLA-B27 is the strongest genetic factor related to familiar aggregation of the disease, we observed that 12% of our HLA-B27-negative patients also had a familial background. As this is much higher than that observed in the general population, these data support the existence of unknown genetic factors other than HLA-B27 that have a role in the familial aggregation of the disease.

We did not observe differences in axial symptoms regardless of the presence or not of HLA-B27. However, we observed a significantly higher frequency of peripheral arthritis, dactylitis, psoriasis, and IBD in HLA-B27-negative AS patients. This association has also been previously described in early forms of AxSpA [5] in which the proportion of HLA-B27-negative patients is much higher than in definite AS patients. Moreover, our data do not support a higher clinical burden of disease in AS patients regarding the presence of HLA-B27. In this sense, HLA-B27-negative AS patients showed significantly higher BASDAI and BASFI scores, even though there were no differences in biologic parameters (ESR and CRP).

Around a fifth of patients included in the analysis had at least one episode of uveitis, this being the most frequent extra-articular manifestation as has been shown in previous studies [17]. Classically, HLA-B27 has been related to the presence of uveitis regardless of the presence or not of a definite AS [18, 19]; thus, in this sense our data are unexpected. However, the results previously reported analyzing the potential association between HLA-B27 in AS patients and the presence of uveitis are quite controversial [7, 9, 13]. It is important to note that 19.4% of HLA-B27-negative AS patients had at least one episode of uveitis. These results, together with the absence of an association between episodes of uveitis and axial inflammation in AS patients, suggest the need for further studies to evaluate the etiopathogenesis of uveitis in AS patients.

Finally, we did not observe any differences in the frequency of coxitis between both groups, in contrast to the data reported by Yang et al. [9]. However, the prevalence of coxitis in our study was low (3.1 and 2.9%, respectively) which makes it difficult to draw any conclusions on this matter.

This study has some limitations. This is a cross-sectional study, and the implications between the observed data and outcomes must be interpreted with caution. Our study did not support a higher percentage of uveitis in the presence or not of HLA-B27. However, we only evaluated the presence or not as a dichotomic measure, and thus we cannot exclude a higher frequency of uveitis flares in AS HLA-B27-positive patients compared with negative patients. The BASRI score was used instead of the mSASSS, which is a more sensitive score to evaluate axial structural changes [20]. Moreover, we do not have data on axial magnetic resonance imaging (MRI), and thus we could not evaluate the influence of bone marrow edema on the potential progression of structural damage. We evaluated only definite AS patients with a long disease evolution (around 8 years) and thus we cannot exclude a potential influence on radiographic progression of HLA-B27 in the early stages of the disease. In this sense, it is not possible to extrapolate these results to nrAxSpA. However, the data observed suggest that, despite HLA-B27 typing, other genetic or environmental factors might play a major role in bone structural damage in AS patients. Finally, given that BASDAI scores were not recorded separately, the Ankylosing Spondylitis Disease Activity score could not be calculated.

## Conclusion

In summary, this is the most extensive study analyzing differences in Caucasian AS patients regarding their HLA-B27 status. This study confirms the previously reported association in AS patients between HLA-B27 and an earlier disease onset and greater family aggregation. However, and interestingly, we do not support an association between HLA-B27 and the extent of axial structural damage, or a higher clinical burden of the disease. Moreover, we cannot prove an association between the presence of anytime uveitis and HLA-B27 in definite AS patients. On the other hand, the absence of HLA-B27 is related, in AS patients, to a higher frequency of peripheral arthritis, dactylitis, and extra-articular manifestations.

## Abbreviations

AS: Ankylosing Spondylitis
AxSpA: Axial spondyloarthritis
BASDAI: Bath Ankylosing Spondylitis Disease Activity Index
BASFI: Bath Ankylosing Spondylitis Functional Index
BASRI: Bath Ankylosing Spondylitis Radiology Index
CI: Confidence interval
CRP: C-reactive protein
ESR: Erythrocyte sedimentation rate
ESSG: European Spondyloarthropathy Study Group
IBD: Inflammatory bowel disease
mSASSS: Modified Stoke Ankylosing Spondylitis Spine Score
nrAxSpA: Nonradiographic axial spondyloarthritis
OR: Odds ratio
