# Correction to: Influence of HLA-B27 on the Ankylosing Spondylitis phenotype: results from the REGISPONSER database

**DOI:** 10.1186/s13075-020-02237-5

**Published:** 2020-06-11

**Authors:** Marta Arévalo, Jordi Gratacós Masmitjà, Mireia Moreno, Joan Calvet, Cristobal Orellana, Desirée Ruiz, Carmen Castro, Pilar Carreto, Marta Larrosa, Eduardo Collantes, Pilar Font, REGISPONSER group, Eduardo Collantes Estévez, Xavier Juanola Roura, Jose Luis Fernández Sueiro, Carlos González Fernández, Jordi Gratacós Masmitjá, Juan Mulero Mendoza, Juan Carlos Torre Alonso, Pilar Fernández Dapica, Mª. Elia Brito Brito Arévalo, Enrique Batlle Gualda, Luis F. Linares Ferrando, Enrique Judez Navarro, Carlos Vázquez Galeano, Teresa Clavaguera Poch, Mª. Cruz Fernández Espartero, Enrique Calero Secall, Manuel Pujol Busquets, Carlos Rodríguez Lozano, Manuel J. Moreno Ramos, Eduardo Cuende Quintana, Manuel Fernández Prada, Rubén Queiro Silva, Estefanía Moreno Ruzafa, Carlos Montilla Morales, Alicia García López, Eugenio Giménez Úbeda, Antonio Juan Más, Cristina Medrano Le Quement, Enrique Ornilla

**Affiliations:** 1 Rheumatology Department, Consorci Corporació Sanitària Parc Taulí, Institut d’Investigació i Innovació Parc Taulí I3PT, Departament de Medicina, Universitat Autònoma de Barcelona, Parc Taulí s/n, 08208 Sabadell, Barcelona, Spain; 2 Rheumatology Department, Hospital General Universitario Reina Sofía/IMIBIC/Universidad de Córdoba, Córdoba, Spain

**Correction to: Arthritis Res Ther (2018) 20: 221**

**https://doi.org/10.1186/s13075-018-1724-7**

Following publication of the original article [1], the authors identified an error in affiliation 1.

The correct affiliation is:

1 Rheumatology Department, Consorci Corporació Sanitària Parc Taulí, Institut d’Investigació i Innovació Parc Taulí I3PT, Departament de Medicina, Universitat Autònoma de Barcelona, Parc Taulí s/n, 08208 Sabadell, Barcelona, Spain.
